# Ovariotomy by Enucleation

**Published:** 1872-12

**Authors:** E. N. Brush


ART. II.—*Ovariotomy by Enucleation.* Report of Cases. By E. N. BRUSH.

The views which are at present held by the profession in regard to the operation of Ovariotomy are in most respects similar, the principal matter in regard to which difference of opinion exists being the treatment of the pedicle.

The questions as to how the pedicle should be secured—whether by clamp or ligature, the material to be used as a ligature if this method is pursued, whether the ligatures should be cut short and the pedicle returned, or they should be brought out of the external incision, or whether the pedicle itself should occupy this position, or clamp and ligature being thrown aside, torsion or the actual cautery should be applied—have all, with many others, been agitating the professional mind ever since the introduction of Ovariotomy as one of the established operations of surgery.

The discovery by Prof. Miner, in 1869, of a mode of procedure by which clamp cautery and ligature were dispensed with, will, without doubt, do much toward settling these questions if, in fact, it does not do so entirely.

The history and *modus operandi* of this method have been sufficiently brought before the profession, and to those who have not been made acquainted with the procedure, it is hoped the report of the following cases will impart some information.

The recent successful application of enucleation in the removal of ovarian tumors has so fully demonstrated its feasibility, if indeed any further demonstration is needed in addition to the successful cases already reported, that it will form sufficient reason for reporting the cases. The first case under consideration was briefly alluded to in a paper on the subject of Ovariotomy recently published by Prof. Miner.* It, however, so clearly illustrates what may be expected by this method that a more complete report may not be out of place in this article.

Case 1.—On the 4th of June last Dr. Miner was calledin consultation with Dr. Mixer of this city to see a patient, aged twenty-three, in whom he had diagnosed an ovarian tumor. After a careful examination, the conclusion arrived at by Dr. Mixer was concurred with, and on consultation with the patient an operation was decided upon. I am indebted to Dr. Mixer for the previous history of the case, which is as follows: Had noticed pains in the right illiac region for about a year; has menstruated quite regularly, but not of usual amount; did not notice any enlargement of the abdomen until about six months ago, since which time the tumor has grown quite rapidly; has never been tapped.

*Operation.*—On Monday, June 17, Dr. Miner proceeded to operate in the presence of Drs. S. F. Mixer, C. C. Wyckoff and Son, E. R. Barnes, and the writer. Dr. Mixer gave chloroform, which was afterward substituted by ether. An incision about four inches in length was made down to the peritoneum, which was divided upon a director. The tumor presented a white, shining appearance, and was found to be attached by its anterior surface to the abdominal walls for a circumference of about nine inches. These attachments were carefully separated, but the walls of the tumor in this locality being exceedingly thin, were accidentally ruptured, and some of the contents escaped. The trocar was immediately plunged in and the contents of the cyst evacuated. The fluid

*American Journal of Medical Sciences, October, 18[illegible]2, page 391.

which was drawn off had a light, chocolate appearance, and readily passed through the canula. The cyst was now lifted from the abdominal cavity, and the pedicle brought to view. It was found to be of medium length, and its vessels were spread out over the walls of the tumor. Dr. Miner introduced his finger into the central portion of the pedicle at its least vascular part, between it and the tumor, and, by a gentle manipulation, the pedicle was separated from the cyst. Two vessels only bled sufficiently to attract attention, and these were easily controlled by torsion. All further hæmorrhage soon ceased on exposure of the pedicle to the air, and the cavity of the abdomen being sponged out it was returned without the application of a single ligature or other mechanical means to prevent hæmorrhage. The slight oozing of blood caused by the separation of the attachments of the tumor to the abdominal walls ceased spontaneously. The wound was now brought together by silver wire sutures, including the peritonæum, supported by adhesive straps, and warm flannel clothes, with bandages, applied over the abdomen. The tumor was multilocular, and weighed eighteen pounds. On recovering ftom the anæsthesia the patient complained of nausea, and vomited frequently. One-fourth grain morphine was directed to be administered every four hours by hypodermic injection; she was also ordered brandy and beef tea. The following is a report of her condition during the time she was seen by Dr. Miner:

June 17, evening—Pulse 120.
" 18, morning—Pulse 108; vomiting in night.
" 19, " " 108; less vomiting; slept.
" 20, " " 98; no vomiting; slept well; urinates without use of catheter; no swelling; no tympanitis; wound healing rapidly.

June 21. Pulse 94; no symptoms of constitutional disturbance; sleeps and eats well.

June 24. Pulse 84.

June 27. Stitches removed; patient well.

During the whole course of recovery from the operation the patient declared that, with the exception of the nausea experienced during the first two or three days, she felt better than at any time just previous to the operation.

To attribute the good result and rapid convalescence in this case entirely to the employment of enucleation in the removal of the tumor would, perhaps, be claiming too much for this mode of operation. It is plain, however, to the most superficial observer that it possesses advantages infinite almost in their application over other methods of procedure, and it cannot but suggest itself to the mind of the reader that the entire absence of all appliances which would tend to excite inflammatory action must have gone far toward assisting in the recovery of the patient.

The pedicle in this case was of medium length—too short, however, to admit its being brought out of the edges of the wound and secured by the clamp without dragging upon the uterus, especially if any portion of it were taken away with the tumor, as is the case when it is severed by the knife in the ordinary operations for ovariotomy. The vessels of the pedicle were large and could be felt to pulsate plainly; to have severed these with the cautery might have controlled the hæmorrhage, it is, however, to be doubted. The silk and metal ligature have been employed successfully in many cases, and have been left in the abdominal cavity without producing any ill effects. Their employment, however, leaves in the cavity of the peritonæum a foreign substance, which in most cases cannot but produce more or less inflammation, and any method which will allow the return of the pedicle to the abdomen free from his source of irritation must meet with approval. The question has been asked, "Can the pedicle be easily separated from the tumor?" In this case just narrated the separation was accomplished with as great facility as attends the separation of cys ic growths from surrounding parts in other portions of the body. The separation was easier, in fact, than was the detachment of the adhesions from the abdominal walls.

Prof. Logan, of the Medical Department of the University of Louisiana, in a private letter, speaking of a recent operation which he performed for the removal of an ovarian tumor, the solid portion of which weighed sixteen a half pounds, says: "I was surprised at the facility with which the enucleation was effected, not a single vessel of sufficient size to throw a jet of blood presented itself, and no ligatures were of course required." This case also had a rapid convalescence, and had not a single bad symptom.

Case 2. Mrs. ———, of Corry, Pa., aged 24, entered the Hospital of the Sisters of Charity, Nov. 1, 1872. Two years ago she first noticed tenderness and swelling in the right illiac region. Since then the growth of the tumor has been gradual, producing but little pain or inconvenience, until the last six months, since when the size of the tumor has increased rapidly, being accompanied by constant and severe pain.

The enlargement was uniform, presenting the appearance of a round, firm, fluctuating tumor. The uterus was drawn upward and to the right, and was distinctly perceptible to the touch upon the outside of the tumor. The tumor had never been tapped. A unilocular cyst of the right ovary was diagnosed, and as the patient had determined to submit to any procedure that might afford relief, an operation was determined upon. Profs. Rochester and H. N. Eastman also saw the patient, and concurred in the diagnosis and propriety of operative interference.

*Operation.*—Wednesday, Nov. 27, Dr. Miner proceeded to operate in the presence of his colleagues of hospital staff and college faculty, the students of the Buffalo Medical College, and various members of the profession. He was assisted by Profs. E. M. Moore, of Rochester; James P. White and M. G. Potter, of Buffalo; and H. N. Eastman, of Geneva.

Prof. Rochester having induced complete anæsthesia with chloroform, which was kept up by ether, an incision about four inches in length was made in the median line down to the peritonæum, which was divided upon a director. Upon opening the peritoneal cavity the tumor came into view. Resting upon its anterior surface and to the right was seen the uterus and fallopian tubes, which were closely adherent to the tumor.

The tumor was found free from attachments to the abdominal parietes, and was therefore easily lifted from the abdomen. A trocar was plunged in and its contents evacuated. The fluid which passed off was dark and viscid and the canula was several times obstructed by what seemed to be masses of fat, bunches of hair also passed through the tube. After having fully evacuated the cyst which was found to be as first diagnosed unilocular in character, Dr. Miner proceeded to carefully separate it from its attachments. These were found to be quite firm, the tumor being closely adherent to the uterus and its ligaments. No pedicle presented itself, and had the application of a clamp or ligature been relied upon, the tumor could not have been removed without encluding the uterus and appendages.

The attachments being all separated, but one vessel was found to bleed, this being caused by the introduction of the trocar through it. The application of torsion being found insufficient to check the hemorrhage, a silver wire ligature was applied, this was cut short and allowed to remain. The cavity of the abdomen was now carefully sponged out, the left ovary was examined and found to be healthy. The wound was now brought together by silver sutures supported by adhesive straps. Warm flannel cloths were applied to the abdomen and the patient returned to bed.

On examination of the tumor, the cyst was found to be thick and composed of layers which were not however closely examined. The fluid contents were dark and thick and contained masses of fat about the size of a filbert and smaller, hair was also found thickly scattered through the fluid. Attached to the inner portion of the cyst was a piece of bone irregular in shape, of about the size of a superior maxilla in an infant; growing from this in different directions were six teeth.

On recovering from the anæsthesia the patient complained of but little pain, but vomited almost continuously. Morphine was ordered by injection. Ice, iced champagne, beef tea &c., were all administered but with no effect, the stomach rejecting every thing.

The patient continued to grow worse and died at 11 o'clock in the forenoon of Dec., 1st, four days after the operation of exhaustion.

No post mortem examination was allowed, and therefore the condition of the pedicle could not be ascertained. There were however no symptoms that indicated internal hemorrhage, and it is presumed that none occurred.

The unfavorable result in this case was hardly unexpected. For a year the irritation produced by the tumor, had provoked distressing nausea, vomiting occurring frequently, as often as twice a day. After the operation this vomiting occurred more frequently, completely exhausting the patient and resulting in death.

The character of the cyst is of no little interest. Dermoid cysts are said to be of slow growth. This was the case for the first eighteen months in the case under consideration, but for the last six months its growth had been rapid and accompanied by severe constitutional disturbance.

Had its true nature been known, and had it produced no more inconvenience than accompanied the first year of its growth, the propriety of an operation might have been questioned. But the rapidity with which the tumor had lately enlarged and the pain and inconvenience which had been distressing the patient, demanded some interference looking toward relief.

The intimate attachment of the tumor to the uterus and appendages, leaving no pedicle to which a clamp or ligature could be applied, offered a test to the feasibility of enucleation, which would severely try it.

The ease with which the tumor was enucleated, without hemorrhage, (the only bleeding vessel of any importance being the one which was torn off by the introduction of the trocar) clearly demonstrated that in cases of this kind, where no other known method could be employed, enucleation was a plan, the application of which was not only imperatively demanded, but was the only one by which the tumor could be removed.

